# Does interpersonal behavior of psychotherapy trainees differ in private and professional relationships?

**DOI:** 10.3389/fpsyg.2015.00765

**Published:** 2015-06-08

**Authors:** Janna I. Fincke, Heidi Möller, Svenja Taubner

**Affiliations:** ^1^University of KlagenfurtKlagenfurt, Austria; ^2^Fachbereich 01 Humanwissenschaften, Institut für Psychologie, University KasselKassel, Germany; ^3^Abteilung Klinische Psychologie, Psychotherapie und Psychoanalyse, University of KlagenfurtKlagenfurt, Austria

**Keywords:** SASB, interpersonal behavior, psychotherapy training, work involvement, relationship settings

## Abstract

**Aim:** The present study aimed to evaluate the effect of trainees’ interpersonal behavior on work involvement (WI) and compared their social behavior within professional and private relationships as well as between different psychotherapeutic orientations.

**Methods:** The interpersonal scales of the Intrex short-form questionnaire and the Work Involvement Scale (WIS) were used to evaluate two samples of German psychotherapy trainees in psychoanalytic, psychodynamic, and cognitive behavioral therapy training. Trainees from Sample 1 (*N* = 184) were asked to describe their interpersonal behavior in relation to their patients when filling out the Intrex, whereas trainees from Sample 2 (*N* = 135) were asked to describe the private relationship with a significant other.

**Results:** Interpersonal affiliation in professional relationships significantly predicted the level of healing involvement, while stress involvement was predicted by interpersonal affiliation and interdependence in trainees’ relationships with their patients. Social behavior within professional relationships provided higher correlations with WI than private interpersonal behavior. Significant differences were found between private and professional relation settings in trainees’ interpersonal behavior with higher levels of affiliation and interdependence with significant others. Differences between therapeutic orientation and social behavior could only be found when comparing trainees’ level of interdependence with the particular relationship setting.

**Conclusion:** Trainees’ interpersonal level of affiliation in professional relationships is a predictor for a successful psychotherapeutic development. Vice versa, controlling behavior in professional settings can be understood as a risk factor against psychotherapeutic growth. Both results strengthen an evidence-based approach for competence development during psychotherapy training.

## Introduction

Psychotherapists’ interpersonal behavior is known to be one of the most important factors for successful psychotherapies. It is assumed that neither theoretical orientation nor level of experience predicts the effectiveness of therapy (Dunkle and Friedlander, 1996; Lambert and Barley, 2001; Beutler et al., 2004; Skovholt and Jennings, 2004; Sandell et al., 2007), but the therapist’s interpersonal skills related to empathy and the ability to foster a warm and attentive relationship with his/her patients (Strupp et al., 1969; Holm-Hadulla, 2000). These behavioral patterns form the therapeutic alliance (Ackerman and Hilsenroth, 2003), which is known to be the most robust predictor for positive therapy outcomes in terms of therapist effects (Lambert, 2013). In order to explore further how interpersonal skills develop during training, we conducted a research project comparing trainees’ private and professional interpersonal behavior. Three major points will discussed in the current paper: (a) are trainees’ experiences of therapeutic success and stress predicted by their social behavior in private or professional relationships (b) do psychotherapy trainees show different behaviors toward patients and significant others and (c) is the therapeutic orientation a distinguishing factor for interpersonal behavioral characteristics and their relation to outcome.

To assess the trainees’ interpersonal behavior, the Intrex questionnaire of the Structural Analysis of Social Behavior (SASB) was used. The SASB operationalizes social behavior along two orthogonal axes describing the level of affiliation or interdependence respectively (Benjamin, 1974). Benjamin (2001) described affiliation as an interpersonal dimension between the poles love and attack, whereas interdependence constitutes an axis from emancipate to control in relation to others. As proposed by Benjamin (1996), it is assumed that specific patterns of social behavior develop during early childhood and will continue to be repeated in future relationships. She divided social behavior into three different focus levels. First, the transitive level, called *parent-like*, describes how individuals identify with attachment figures and behave like the significant other (identification). Second, the intransitive level, called *child-like*, describes how a person responds to his/her parent’s actions and later behaves as if he/she was in control if the significant other (recapitulation). And on the third level, the child internalizes the way his/her parents treated him/her, which in turn shapes his/her behavior toward him/herself in the future (introjection; Benjamin, 1996). The terms introjection and identification originate from psychoanalytic (PA) theory and relate to varying degrees of internalizing aspects of significant others (Sandler and Perlow, 1989). In this paper, the terms introjection and identification will be used following the conceptualization by Benjamin (2001). Simpson and Rholes (1998) pointed out that early childhood experiences with parents have a major impact on a person’s way to relate to other individuals, and also affects the therapists’ alliance to their patients (Mallinckrodt and Nelson, 1991). Henry and Strupp (1994) reported, that the therapists’ perceived quality of alliance toward their clients is affected by the early relationships to their parents. Furthermore, this perception of alliance was associated with therapeutic outcome. In addition to the former study, Hilliard et al. (2000) found a significant correlation between the therapists’ and patients’ affiliation in relationships during early childhood and the therapeutic success. However, the relational history predicted the perception of the therapy process for therapists and patients separately, meaning that a therapist with a more affiliative early relationship to his/her parents does not influence the patient’s experience of effective therapy, but does affect the therapist’s own perception of his/her therapeutic effectiveness. Henry et al. (1986) observed more specific effects of the therapists’ interpersonal styles on the patients’ introject. The research group found that the psychotherapist’s level of affirmation, understanding, helping, and protecting increases the probability of a good outcome in therapy. In contrast, therapists with hostile and more controlling social behaviors, showed less success in their therapeutic work (Henry et al., 1986). In a further study Lukei (2004) used the SASB Intrex short-form questionnaire (Benjamin, 1988) to evaluate the improvement of the therapeutic relationship over time. For this purpose, she analyzed the therapist’s interpersonal and the patient’s introject level of affiliation. Results showed, that the patient’s affiliation increases during short-term psychotherapy in correlation with the therapist’s level of affiliation. These findings stress the importance of interpersonal skills for the therapeutic alliance and the quality of operationalization of the SASB measures.

According to Orlinsky and Rønnestad (2005) social behavioral qualities build the groundwork and maintain the therapist’s therapeutic work involvement (WI). The authors conceptualized two developmental cycles of WI based on findings of a cross-sectional study of the Society of Psychotherapy Research’s (SPR) Collaborative Research Network (CRN). Higher levels of healing involvement (HI) characterize the positive cycle. If HI dominates psychotherapists’ experience, their work is effective and they are motivated as well as open for affirming relationships with their patients. To reach this effective state, it is important that therapists perceive themselves in a state of professional growth, which improves their satisfaction with their work and is connected to a positive work moral. This leads to optimism about their therapy outcomes and a feeling that they are doing valuable work. In contrast, the negative cycle is related to therapists’ stressful involvement (SI). Here, low professional growth results in psychotherapists’ feelings of insecurity and inflexibility within their therapeutic work. Thus, doubts about positive therapy outcomes increase, while motivations to receive new theoretical inputs or attend supervisions decrease. Due to the fact that psychotherapists within the negative cycle cope by avoidance, there is a high risk of stagnation (Orlinsky and Rønnestad, 2005). Model-confirming data from longitudinal assessment of psychotherapist development after training does not exist. Concerning trainee development, Taubner et al. (2013) presented data from a longitudinal study that showed an increase in HI that was correlated to the level of introject affiliation, i.e., how forgiving and loving or harsh they treat themselves. These correlations were moderated by the satisfaction with the trainee’s personal therapy. However, the impact of interpersonal behavior in professional and private settings on WI remains unclear for psychotherapists and especially for trainees.

Among others, Rønnestad and Skovholt (2003) suggested that psychotherapists’ therapeutic competences are grounded in private relational experiences. According to the authors, already before starting training, psychotherapists function as ‘lay helper’ within their social environment. In this special role, individuals behave on the basis of their interpersonal styles including a strong sympathy for their relatives and friends in order to support them in resolving emotional difficulties. With the beginning of a professional training, trainees realize that they cannot fully rely on these private techniques anymore and have to adapt to the new therapeutic approach. Therefore, many trainees experience this period as a challenge but feel enthusiastic at the same time. At this stage of development, they are highly sensitive to criticism by their supervisors and patients, which is accompanied by self-doubts and an increased level of stress. With on-going training, trainees develop more self-confidence within their work; however, they still feel a high level of dependence from their supervisors. After finishing training, moderated by a growth of experiences, psychotherapists feel more secure within their work and experience a higher trust toward their personal assumptions. Nevertheless, the development of psychotherapists is a life-long process (Rønnestad and Skovholt, 2003). In further retrospective interviews with psychotherapists, the authors were able to identify a tendency that distressing events in the private lives of psychotherapists can affect their development negatively in short term, but often positively in the long term. Therefore, Rønnestad and Skovholt (2003) suggested an association of a therapist’s professional and private life. Nissen-Lie et al. (2013) could identify a relevant link between a therapist’s satisfaction in personal life and their ability to develop an efficient alliance to their patients. At the moment, little is known about the relationship between social behaviors in psychotherapy trainees’ professional and private lives. However, it can be assumed that the therapeutic social behavior in the beginning of training is affected by experiences in private relationships. Hersoug et al. (2009) suggested that therapists’ relationships in private life have a relevant impact on the capability to develop an enriching relationship to their patients. The authors found that therapists who experience themselves as reserved within personal relationships, were less likely to develop a positive alliance with their patients. These findings address experienced psychotherapists only; currently no published studies exist evaluating the interpersonal behavior of psychotherapists in training.

Consequently, there are no studies on the question of how different therapeutic schools may support differing interpersonal behavior during training. Taubner et al. (2010) found that psychotherapy trainees with different theoretical approaches vary significantly in their therapeutic attitudes already at the beginning of training. Attendants of a cognitive behavioral therapy (CBT) training rated *support* as the most important attitude and *adjustment* as the main curative factor for their therapeutic work, whereas for trainees in PA, Psychodynamic (PD), and Psychotherapy *insight* was the most relevant curative factor and *neutrality* the most important therapeutic stance. According to Clarici (2015) therapeutic stance bases on the individual’s private and professional roles and is defined by his/her strategies to deal with mentally challenging material. Hence, the preferred stance, as therapeutic technique of choice, is not necessarily stable over time, but can be redefined to some extent, when the therapist realizes that he/she has to modify his/her chosen method to make it more suitable to the patient’s problem (Sandler, 1992). However, findings of specific school related therapeutic attitudes (cf. Taubner et al., 2010) might be associated with well-known differential basic approaches of these therapeutic orientations. For example PD and PA represent approaches focusing on sub-conscious and unconscious processes. Freud (1915) defined the consciousness as an immediate descriptive perception within a latent sequence of time. Consequently, the subconscious refers to a latent lack of consciousness, however, the individual is still able to access information’s of these situations retrospectively. Unlike these, the unconsciousness hides psychic contents in the name of repression. Within PA the therapists work on a patient’s intrapsychic conflicts using his/her emotional reactions. PD is guided by specific goals the patient tries to achieve within the therapy in contrast with PA, where interpersonal and intrapsychic conflicts are the focus of the therapy (Neukom et al., 2011). In opposition, CBT works with the consciousness of the patients. During therapy, a CBT therapist concentrates on the modification of dysfunctional cognitions and behavioral patterns in order to solve current psychopathological symptoms (Reinecker and Lakatos-Witt, 2011). Safran and Muran (2000) stress the connection between therapeutic methods and specific social acting. Also by referring on them, it can only be assumed that trainees of various theoretical orientations differ in their interpersonal professional behavior, since no evaluation of interpersonal behavior of psychotherapy trainees has been published yet. Private interpersonal behavior could be related to self-selection processes when choosing a therapeutic approach. Taubner et al. (2014) found indications that therapeutic attitudes, interest for mentalization but also the degree of openness for new experiences influence the choice of a specific psychotherapeutic school. These results indicate that personality related traits may interact with trainees’ social behavior derived from private relationships and both may influence their choice of therapeutic school.

In order to explore trainees’ interpersonal behavior, both private and professional, as well as their impact on the psychotherapeutic development, the present study will focus on the following questions:

Question 1: How much impact does interpersonal behavior in private or professional relationships have on WI in psychotherapy trainees?

We assume that trainees’ variance of WI can be explained by their interpersonal behavior in two ways. Firstly, interpersonal behavior influences WI directly by means of a good match between task and abilities. Secondly, good relational skills may lead to better outcomes, which in turn have positive effects on WI. Therefore, we presume that the level of affiliation in professional and private relationships predicts the trainee’s HI significantly (cf. Taubner et al., 2013). Furthermore, we expect that trainees, who show more controlling and less affiliative behavior, tend to have higher levels of SI.

Question 2: Do trainees differ significantly in interpersonal behavior among professional and private relationships?

Rønnestad and Skovholt’s (2003) developmental model suggested that in the beginning of training, trainees rely on their interpersonal experiences derived from private relationships. Hence, we assume that no significant interpersonal differences in trainees’s social behavior in private and professional settings can be found.

Question 3: Do significant differences exist in trainees’ social behavior concerning private and professional relationships between therapeutic school orientations?

Different tasks and therapeutic attitudes in therapeutic approaches could lead to different interpersonal behavior in professional relationships. In detail, we hypothesize that CBT trainees show a higher level of controlling behavior based on their active approach to modify cognitive structures using therapeutic attitudes, like support and adjustment (cf. Taubner et al., 2010). Based on their theoretical approach, we assume that PA and PD trainees grant more autonomy toward their patients than CBT trainees, demonstrated by the preference of the therapeutic attitude insight and low levels of adjustment (cf. Taubner et al., 2010). As previous studies suggested, successful therapies are dependent on the therapist’s interpersonal affiliation, but independent from the psychotherapist’s theoretical orientation (Lambert and Barley, 2001). Therefore, we propose that no significant differences in the level of interpersonal affiliation can be found between the trainees of various theoretical orientations. Furthermore, due to self-selection-processes, trainees of different therapeutic approaches may also differ in their interpersonal behavior in private relationships. This has not been investigated before and this is why we have no hypothesis on how trainees might differ.

## Materials and Methods

### Procedure

For the current study, data from two samples have been collected. The first sample was assessed in the DFG-founded research project ‘Competence Development of Psychotherapy Trainees’ (Prof. Dr. Heidi Möller, University of Kassel, Germany; Prof. Dr. Svenja Taubner, Alpen-Adria-University, Austria). The data acquisition took place in Germany from March 2011 until July 2012. In total, 29 requests were sent out to psychotherapy training institutions. A total of 17 training institutes confirmed their participation in the study (PA: nine; CBT: two; Integrative Institutes: four). Institutes were chosen for their psychotherapy orientations without known systematic distortions between the attending and non-attending institutions. Participants received an expense allowance of 100 Euros. The participants were informed about the study verbally as well as in written form. If they agreed, they were asked to give informed consent. In total, 184 trainees participated in the study with an average of 10.44 per training institution (SD: 12.79), which equals 25.21 per cent of the total number of trainees within the first four semesters of psychotherapy training in the participating institutes. Some assessments of specific test instruments took place at the training institute, which will not be part of the current analysis. Questionnaires were answered in online surveys. The total sample size, used for present study varies within the test variables, due to missing values. Nine missing values were found within the Work Involvement Scale (WIS) variables HI and SI (*N* = 175) as well as one missing value in the variable semester (*N* = 183).

The second sample was collected within the research project *Therapeutic Identity in Psychotherapy Training* from June 2006 until August 2007. For acquisition; 32 private and university-affiliated German training institutes were randomly selected from a list of 148 institutes. All of them were oriented toward PA, CBT, or PD. A decline in cooperation was given by CBT and PD institutes exclusively. Twenty five state-certified institutes confirmed their participation. The main reason for non-participation was other research projects. In total, 700 questionnaire sets were sent out to the institutes. The trainees received the questionnaires either by email, or by a class hand-out, depending on the institutes’ enrolment procedure. A voluntary and anonymous participation was insured to each participant. A total of 171 participants completed the questionnaire sets, which equals a response rate of 24% (23% of PA institutes, 27% of PD institutes, 22% of CBT institutes). More detailed evaluations could not be conducted based on the distribution and return procedures. However, the survey’s response rates corresponded with the generic response rate in German psychotherapy studies (Strauß et al., 2009). An exclusion of 36 participants followed, because they did not answer substantial sections of the questionnaires (>20%) or missed out entire subscales. As a result, the sample size decreased to a total of 135 participants. Within the variable ‘semester’ another four missing values were identified, which equals a sample size of *N* = 131.

### Sample

#### Characteristics of Sample 1

**Table 1** shows a detailed overview of the sample structure. In Sample 1 trainees’ average age was 31.4 years with a significant age difference between PA and CBT trainees [*F*(2,183) = 5.600, *p* = 0.004]. The sex distribution shows that significantly more women (84.2%) than men (15.8%) participated in the study (χ^2^ = 0.45, *p* = 0.798). The participants’ duration of training differed between theoretical orientations, especially between CBT and Psychoanalysis (CBT: *M* = 1.8; PD: *M* = 2.0; PA: *M* = 3.5). Participants in CBT training studied significantly less semesters than PA trainees [*F*(2,182) = 15.8, *p* = 0.000]. However the distribution of training semesters varies the most in the group of PA orientated participants (CBT: SD = 1.1; PD: SD = 1.4; PA = 2.3).

**Table 1:** Results of descriptive analyses.

	Theoretical orientation^a^			Total	Test
	CBT	PD	PA		
**Sample 1 professional setting**
Sample Size (%)	65 (35.3)	80 (43.5)	39 (21.2)	184 (100)	
**Sex (%)**
Female	54 (83.1)	69 (86.3)	32 (82.1)	155 (84.2)	χ^2^ = -0.45
Male	11 (16.9)	11 (13.8)	7 (17.9)	29 (15.8)	
**Age**
Range	28–31	30–33	32–36		*F*(2,183) = 5.6^∗^
M (SD)	29.6 (5.31)	31.8 (7.14)	33.8 (7)	31.4 (6.7)	
**Semester**
Range	1.5–2.0	1.7–2.3	2.7–4.3		*F* (2,182) = 15.8^∗∗^
M (SD)	1.8 (1.1)	2.0 (1.4)	3.5 (2.3)	2.3 (1.7)	
**Sample 2 private setting**
Sample size (%)	40 (29.6)	41 (30.4)	54 (40)	135 (100)	
**Sex (%)**
Female	36 (90.0)	31 (75.6)	40 (74.1)	107 (79.3)	χ^2^ = 4.0
Male	4 (10.0)	10 (24.4)	14 (25.9)	28 (20.7)	
**Age**
Range	31–36	34–38	39–42		*F*(2,134) = 13.37^∗∗^
M (SD)	33.4 (6.8)	36.1 (5.8)	40.4 (7.2)	37 (7.3)	
**Semester**
Range	4–6	6–8	8–10		*F*(2,134) = 13.06^∗∗^
M (SD)	4.6 (3.1)	6.9 (3.7)	9.0 (5.0)	7 (4.5)	

*^a^CBT, cognitive behavioral therapy; PD, psychodynamic therapy; PA, Psychoanalytic therapy. ^∗^p < 0.05, ^∗∗^p < 0.01.*

#### Characteristics of Sample 2

In Sample 2 all participants attended part-time training with a minimum duration of 5 years. As **Table 1** shows, the participant’s duration of training at the time of acquisition varied from one to 30 semesters (*M* = 7; SD = 4.5). A training duration of over 5 years was given in 24% of the cases. Participants of the CBT group were significantly shorter in training than PA trainee’s [*F*(2,134) = 13.06; *p* = 0.000]. Significantly more women participated, with no significant differences between the psychotherapeutic orientations (χ^2^ = 4.02; *p* = 0.13). At the time data was collected, the average trainee’s age was 37 years (SD = 7.3) with significant age difference between CBT and PA trainees [*F*(2,134) = 13.37; *p* = 0.000]. In this sample four missing values were found in ‘duration of training’ (*N* = 131). Also within the variables HI and SI 11 missing values were detected (*N* = 124).

#### Comparison of the Samples

The samples differ significantly in age and semester (**Table 2**). Trainees in the second sample are significantly older (*t* = 7.13; *p* = 0.00) and longer in training than persons of sample 1 (*t* = 13.25; *p* = 0.00). Trainees’ age and duration in training correlate significantly (*r* = 0.48; *p* = 0.00) therefore, only age was used as covariate in analyses comparison both the samples. Sex distributions between both samples provided no significant differences (χ^2^ = 1.32; *p* = 0.25).

**Table 2:** Comparison of Sample 1 and 2.

	Means of Sample 1	Means of Sample 2	Test
Sex			χ^2^ = 1.32
*Female*	155	107	
*Male*	29	28	
Age	31.4	37	*r* = -0.37^∗∗^
Semester	2.3	7	*r* = -0.60^∗∗^

*^∗^*p* < 0.05, ^∗∗^*p* < 0.01.*

### Instruments

#### Interpersonal Behavior

To evaluate trainees’ interpersonal behavior, the Intrex short-report based on the Structural Analysis of Social Behavior (SASB) by Benjamin (1983) was used. The SASB circumplex model is based on two-dimensional variables “Affiliation” and “Interdependence,” located in the center of three circumplex surfaces. The first surface is the *transitive* level, which describes actions toward other people (e.g., the patient/significant other). The *intransitive* surface records reactions to other person’s behavior, therefore the focus is on the other person. The third surface is the *introject*, which focuses on the individual’s self-representation. In this study, we combined the data from the transitive and intransitive levels in order to receive an interpersonal dimension. On the transitive level, affiliation scores from *attack* to *love.* Complement reactions on the intransitive level reach from *recoil* to *love.* The Interdependence scale is located vertically from the affiliation dimension. On its transitive level it scores from *emancipate* to *control* complementary to the intransitive level, which describes the reactional behavior from *separate* to *submit.* All scales were calculated using Pincus et al. (1998) vector-formula. The Intrex short-version questionnaire includes 80 items, each of which is answered on a scale from 0 to 100, we instead transformed this ranking into a scale from 0 (“not at all true”) to 6 (“completely true of myself”). For example the participants were asked to rate the following statement on the transitive level “I let him speak freely, and warmly try to understand him even if we disagree.” On the intransitive level the item is modified in a reactive way, for example: “He lets me speak freely, and warmly tries to understand me even if we disagree.” The reliability on the interpersonal level shows a good internal consistency in affiliation (Cronbach’s α = 0.823) as well as in interdependence (Cronbach’s α = 0.654). In the present study the introject outcome is not included.

#### Work Involvement Scales

The trainees WI was evaluated by using the WIS developed by Orlinsky and Rønnestad (2005). This short self-report offers psychotherapists a reflection on their state of mind during therapeutic work. The WIS was developed based on findings by the SPR CRN. In those the *Development of Psychotherapist Common Core Questionnaire* (DPCCQ, Orlinsky et al., 1999) was applied to evaluate the development of more than 10.000 psychotherapists worldwide. Using a factor-analysis two subscales were identified for the WIS; HI and SI. HI comprised 25 items, whereas SI involves 22 items. Both subscales can be answered on either a three- or five-point rating scale. For example, “How effective are you at engaging patients in a working alliance?” which can be answered on a scale from 0 (never) to 5 (very often). Another example is, “Currently, how often do you feel demoralized by your inability to find ways to help a patient?” with answers from 0 (not at all) to 3 (very much). HI measures therapeutic relational skills like being accommodating, invested and affirming. SI includes attributes like anxiety, boredom and copying by avoiding. The internal consistence of both scales is satisfying (HI: Chronbach’s α = 0.76; SI: Chronbach’s α = 0.66).

### Data Analyses

The Statistical Package for the Social Science (SPSS) version 21.0 was used to analyze the data. Regarding to the hypotheses, the analyses were divided into two sections.

To evaluate the first question, we analyzed, if trainees’ WI is explained by their interpersonal behavior in private as well as professional relationships. For this purpose, we analyzed the data in two hierarchical multiple-regression analyses. Each WIS subscale, HI and SI, were used as dependent variable. The Intrex subscales affiliation and interdependence were used as independent variables in order to examine, how much of the total variance of either HI or SI can be explained by the individual’s social behavior in professional and private relationships. The analyses were divided in three steps. In the first step, exclusively the covariate variables age and sex were inserted as independent variables. Followed by a multiple regression including the both main factors affiliation and interdependence. In the last step the relational setting was added in order to proof whether the type of relationship has got an impact on the WI.

Regarding the second and third question, we assumed that the trainees’ social behavior is independent from the setting of interaction (private vs. professional; question 2), but dependent from their psychotherapeutic orientation (question 3). The trainee’s interaction in professional (Sample 1) and private relationships (Sample 2) was compared in a two-factor-covariance analysis. The first factor was setting of relationship; the second factor was theoretical orientation (CBT vs. PD vs. PA) whereas; affiliation respectively. interdependence was used as independent variables. In the following analyses variables, which showed significant correlations with affiliation and interdependence, were controlled as covariates.

The data sets were modified according to the propositions of Fidell and Tabachnick (2003). In accordance with their suggestions, the data samples were corrected if variables showed data points with an absolute *z*-value score >2.5. Those specific item raw scores were adjusted to an absolute *z*-value of 2.5 manually. In Sample 1, the Intrex variable affiliation showed four scores with an absolute *z*-score > 2.5; in interdependence five scores were identified. Within the WIS subscales, HI and SI, three scores in each variable were observed and corrected. In Sample 2 interdependence (Intrex) and HI (WIS) had two cases of absolute *z*-scores > 2.5.

## Results

### Descriptive Analyses

**Tables 3** and **4** provide the correlations of age, sex, and semesters of training with the main variables mentioned above (affiliation, interdependence, HI and SI). The correlations were different between the two samples. The scale interdependence correlates with the duration of training in the professional setting only (*r* = 0.17; *p* = 0.02). Affiliation in private relationships correlated negatively on a significant level with the age of the trainee’s. Whereas in Sample 1 age showed a positive relation with HI (*r* = 0.18; *p* = 0.02) and were negatively associated with SI (*r* = -024; *p* = 0.00). However, age and the duration of training show a positive correlation in each of the samples (Sample 1: *r* = 0.24, *p* = 0.00; Sample 2: *r* = 0.47, *p* = 0.00). Hence, only age was used as possible confounder to prevent a decrease of test power. Significant sex differences could be observed in both samples in interdependence, HI (*r* = 0.30; *p* = 0.00) and SI (*r* = -0.27; *p* = 0.00). Only in Sample 2, affiliation in private relationships differed significantly between the trainee’s sexes (*r* = 0.48; *p* = 0.00). Therefore, sex was included as covariate in the following main analyses.

**Table 3:** Descriptive analyses of Sample 1.

Measure	*N*	*M*	SD	Age	Semester	Sex
(1) Affiliation	184	120.73	73.99	0.124	-0.04	-0.08
(2) Interdependence	184	52.58	39.41	0.135	0.17^∗^	0.45^∗∗^
(3) HI	175	10.38	1.15	0.18^∗^	0.13	-0.15^∗^
(4) SI	175	4.71	1.61	-0.24^∗∗^	-0.04	-0.37^∗∗^
(5) Semester	183	2.25	1.68	0.24^∗∗^		0.11

*For the correlations, interpersonal affiliation, interpersonal interdependence, HI and SI were centered at their means. ^∗^*p* < 0.05, ^∗∗^*p* < 0.01.*

**Table 4:** Descriptive analyses of Sample 2.

Measure	*N*	*M*	SD	Age	Semester	Sex
(1) Affiliation	137	170.39	83.61	-0.22^∗∗^	0.11	0.48^∗∗^
(2) Interdependence	137	90.82	50.06	-0.15	-0.07	0.18^∗^
(3) HI	140	11.04	1.16	0.05	0.15	0.30^∗∗^
(4) SI	139	5.28	1.46	-0.12	-0.20^∗^	-0.27^∗∗^
(5) Semester	152	7.10	4.45	0.47^∗∗^	1.0	0.01

*For the correlations, interpersonal affiliation, interpersonal interdependence, HI and SI were centered to their means. ^∗^p < 0.05, ^∗∗^p < 0.01.*

### Social Behavior as Prediction of Work Involvement

For the analysis of the social behavior’s impact on WI hierarchical multiple-regression analyses were conducted. To insure that no autocorrelation exist between the residuals a *Durbin–Watson-Test* was used. If the test showed a significant result, further analysis via scatterplot and correlations of the non-standardized residuals followed to secure a non-systematic correlation between the residuals. The test requirement of homoscedasticity was proved by another scatterplot with a positive result (Janssen and Laatz, 2013). Furthermore, the *Variance Inflation Factor (VIF)* as well as the *Tolerance* test could exclude multicollinearity for the present analyses (Bühner and Ziegler, 2009). For this calculation, a negative result of the Shapiro–Wilk-Test was also solved by the central limit theorem a violation (Tabachnick and Fidell, 2013).

In both multiple regressions, the trainee’s sex and age were included as covariate variables in order to control their impact on the independent variables, SI and HI. Results of the analyses show that affiliation predicts a significant part of HI’s total variance (Δ*F*(4,298) = 13.84; β = 0.21; *p* = 0.00; ΔR^2^ = 0.15) whereas; the level of interdependence in social behavior has no significant impact on HI. Also an interaction model was observed by comparing both relational settings [Δ*F*(5,298) = 15.00; β = -0.23; *p* = 0.00; ΔR^2^ = 0.19]. This result shows, that trainees’ interpersonal behavior in professional relationships predicts significantly more variance of HI (see **Table 5**).

**Table 5:** Summary of hierarchical regression analysis for interpersonal behavior with relational setting predicting healing involvement.

Variable	*B*	SE	β	ΔR^2^
Step 1: HI and covariates				0.08^∗∗^
Sex	-0.58	0.15	-0.23^∗∗^	
Age	0.03	0.01	0.24^∗∗^	
Step 2: Main predictors				0.15^∗∗^
Interpersonal affiliation	0.21	0.06	0.21^∗∗^	
Interpersonal interdependence	0.10	0.06	0.10	
Step 3: Interaction terms				0.19^∗∗^
Interpersonal affiliation × interpersonal interdependence × relational setting	-0.47	0.11	-0.23^∗∗^	


*HI, interpersonal affiliation and interdependence were centered at their means. N = 299, ^∗^p < 0.05, ^∗∗^p < 0.01.*

In a direct model the trainee’s level of interpersonal affiliation and interdependence predicts SI significantly [affiliation: Δ*F*(4,298) = 14.60; affiliation: β = -0.24; *p* = 0.00; interdependence: β = -0.20; ΔR^2^ = 0.15). In comparison to HI, the interpersonal variables show a negative association with the trainee’s stress level. Hence, less affiliative and less autonomy-granting behaviors are associated with SI. Interpersonal behaviors in private and professional relationships affect the level of SI significantly different. The trainee’s social behavior in professional relationships predicts significantly more variance of SI and HI [Δ*F*(5,298) = 16.191; β = -0.25; *p* = 0.00; ΔR^2^ = 0.20; see **Table 6**].

**Table 6:** Summary of hierarchical regression analysis for interpersonal behavior with relational setting predicting stressful involvement.

Variable	*B*	SE	β	ΔR^2^
Step 1: SI and covariates				0.03^∗∗^
Sex	0.39	0.15	0.15^∗^	
Age	-0.02	0.01	-0.12^∗^	
Step 2: Main predictors				0.15^∗∗^
Interpersonal affiliation	-0.24	0.06	-0.24^∗∗^	
Interpersonal interdependence	-0.20	0.06	-0.20^∗∗^	
Step 3: Interaction terms				0.20^∗∗^
Interpersonal affiliation and interdependence × relational setting	-0.50	0.11	-0.25^∗∗^	

*SI, interpersonal affiliation and interdependence were centerd at their means. N = 299, ^∗^p < 0.05, ^∗∗^p < 0.01*

### Comparison of Trainee’s Interpersonal Behavior in Psychotherapeutic Orientation and Relational Setting

**FIGURE 1:**
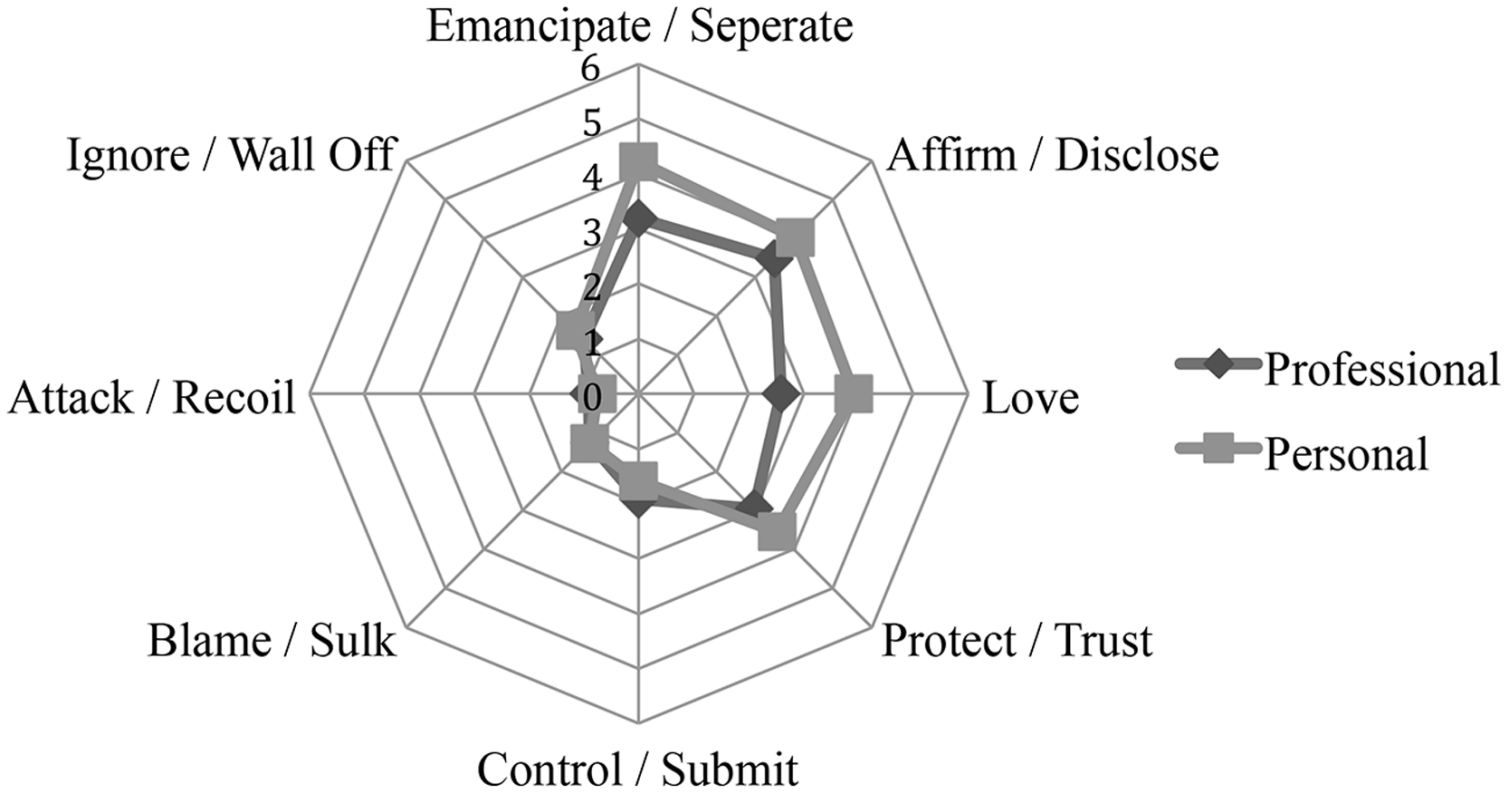
**Interactional circumplex model comparing trainee’s social behavior in private and professional relationships**. The graph illustrates the eight-item Structural Analysis of Social Behaviour (SASB) surface of the Intrex short form questionnaire. Points demonstrate the mean values in each item on transitive and intransitive level.

The following graph illustrates a comparison of trainees’ interpersonal characteristics in private and professional relationships using the SASB eight-item Intrex surface (see **Figure 1**). The figure collapses the interactional behavior on the transitive and intransitive level. Significant differences of trainees’ social behavior in professional and private relationships were found for each item, with the exception of the items ‘blame/sulk’ and ‘attack/recoil’ (see **Table 7**).

**Table 7:** Comparison of private and professional relationships in transitive and intransitive Intrex items.

Variable	*n*	M	SD	DIFF	df	*t*	*p*	*d*	*1-*β
**Emancipate/Separate**									
*Professional*	184	3.19	0.73	0.95	317	11.31	0.00	1.0	1.0
*Private*	135	4.14	0.75						
**Affirm/Disclose**									
*Professional*	184	3.49	0.71	0.51	317	5.90	0.00	1.0	1.0
*Private*	135	4.00	0.84						
**Love**									
*Professional*	184	2.59	0.88	1.32	317	13.74	0.00	1.0	1.0
*Private*	135	3.92	0.81						
**Protect/Trust**									
*Professional*	184	2.96	0.70	0.64	317	7.27	0.00	1.0	1.0
*Private*	135	3.60	0.86						
**Control/Submit**									
*Professional*	184	1.92	0.83	-0.32	317	-3.48	0.00	1.0	1.0
*Private*	135	1.60	0.77						
**Blame/Sulk**									
*Professional*	184	1.26	0.75	0.11	317	1.21	0.23	1.0	1.0
*Private*	135	1.37	0.81						
**Attack/Recoil**									
*Professional*	184	0.91	0.78	-0.10	317	-1.16	0.25	1.1	1.0
*Private*	135	0.80	0.77						
**Ignore/Wall off**									
*Professional*	184	1.40	0.76	0.23	317	2.59	0.01	0.29	0.82
*Private*	135	1.63	0.79						

Trainees’ differences in social behavior were tested in a two-factorial covariance analyses (ANCOVA). Variables had to provide a normal distribution, which was tested by the *Shapiro–Wilk-Test*. According to the central limit theorem, a violation of this test requirement could be ignored in the present sample (degree of freedom >20). As further requirement, variance homogeneity could not be found for affiliation; therefore the level of significance was reduced from 0.05 to 0.01 in the following analyses (Tabachnick and Fidell, 2013).

**FIGURE 2:**
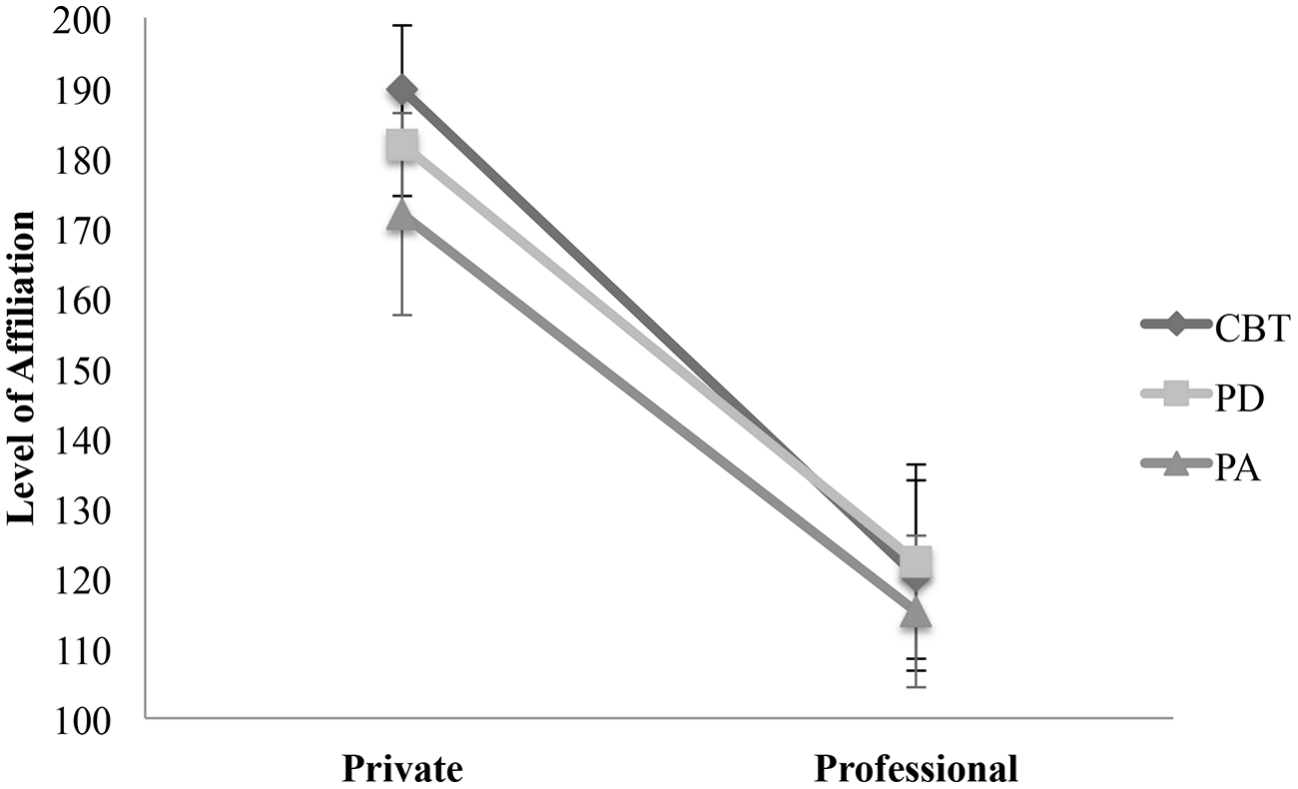
**Comparison of affiliation between therapeutic orientation within private and professional relationships**. The points illustrate mean values of affiliation in private and private and professional relationships. Private – CBT: *M* = 189.72, SD = 86.12; PD: *M* = 181.90, SD = 88.70; PA: *M* = 171.96, SD = 79.54. Professional – CBT: *M* = 120.35, SD = 121.42; PD: *M* = 122.33, SD = 66.01; PA: 115.24, SD = 90.05.

The covariance analysis showed a significant higher affiliative behavior in private than in professional relationships [*F*(5,318) = 40.908, *p* = 0.000, η_p_^2^ = 0.116]. This was independent from theoretical orientation and interaction between setting of relationship and therapeutic school [Theoretical Orientation: *F*(5,318) = 0.477, *p* = 0.615, η_p_^2^ ≤ 0.001; Setting*Theoretical Orientation: *F*(5,318) = 0.152, *p* = 0.859, η_p_^2^≤ 0.001; see **Figure 2**).

**FIGURE 3:**
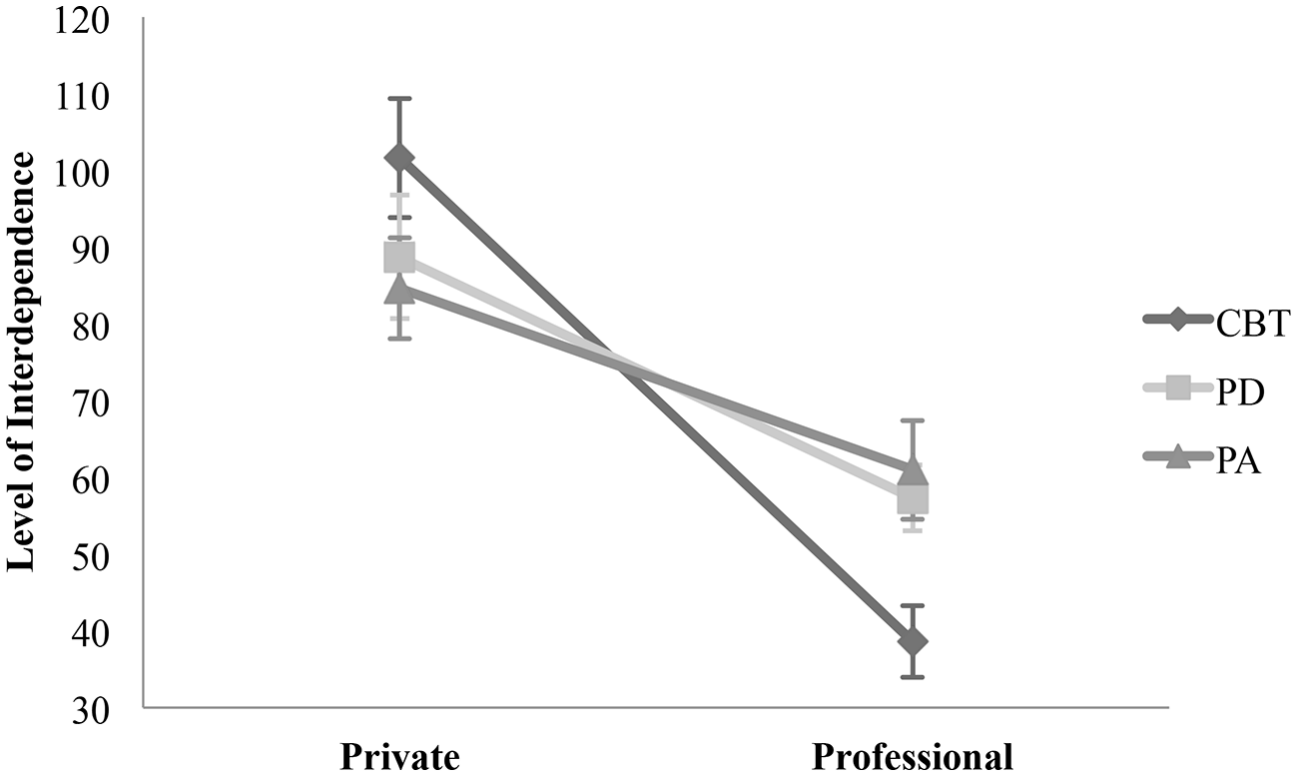
**Comparison of interdependence between therapeutic orientation within private and professional relationships**. The points illustrate mean values of interdependence in private and private and professional relationships. Private – CBT: *M* = 101.62, SD = 7.77; PD: *M* = 88.74, SD = 8.04; PA: *M* = 84.66, SD = 6.58. Professional – CBT: *M* = 38.60, SD = 4.67; PD: *M* = 57.32, SD = 4.27; PA: 60.97, SD = 6.43.

The analysis of interdependence revealed more controlling behavior in professional than in private relationships across all orientations [*F*(5,318) = 53.356, *p* = 0.000, η_p_^2^ = 0.146]. However, participants in CBT training allow the greatest amount of autonomy in private relationships (CBT: *M* = 99.50; PD: *M* = 88.65; PA: *M* = 86.38) and the highest level of controlling (low interdependence) behavior in professional settings (CBT: *M* = 38.78; PD: *M* = 57.40; PA: *M* = 60.97). This outcome results in a significant interaction between theoretical orientation, type of psychotherapy training and the type of relationship [*F*(5,315) = 4.691, *p* = 0.010, η_p_^2^ = 0.029]. **Figure 3** illustrates this result by an intersection of the type of training and interdependence in professional and private relationships. No significant difference between the trainees’ theoretical orientation and their level of interdependence were found in each of the relational settings [*F*(5,318) = 0.274, *p* = 0.761, η_p_^2^ = 0.002].

## Discussion

The present study aimed to answer the questions of how interpersonal behavior of psychotherapy trainees’ influences therapeutic growth and how trainees’ private relationships and professional therapeutic relationships differ in terms of social behavior. To explore these questions, we formulated three sub-questions, which will be discussed in the following.

First, we analyzed if trainees’ interpersonal behavior in both relational settings had an impact on the WI. We found that the trainees’ level of affiliation and interdependence in professional relationships was a significant predictor for WI. Results showed that higher levels of interpersonal affiliation in professional relationships were associated with higher levels of HI during training. Whereas, lower levels of affiliation and higher levels of control in professional relationships were associated with higher levels of SI. These results led us to the assumption that trainees who behaved more affirmative, loving, and protective were more optimistic regarding their professional growth. On the other hand a distanced and less spontaneous interaction style provoked trainees’ dissatisfaction, presumably because of missing therapeutic success. Trainees’ interpersonal behavior in private relationships seemed to have a lower impact on the variables HI and SI. When predicting the variance of SI, both affiliation and interdependence in private settings had a significant impact. A negative correlation between these variables made us emphasize that controlling interpersonal behavior and a lower degree of affiliation were associated with a higher level of SI. Meaning, if a person interacted in a more hostile and controlling manner with a significant other, there was a higher chance of feeling pessimistic about the psychotherapeutic work; however, the professional interpersonal pattern proved more important in relation to the quality of professional growth.

Secondly, we asked whether trainees’ social behavior differs in private and professional relationships. Results showed that trainees’ interpersonal social behavior was significantly different in private and professional settings. When asked to describe their professional relationships with their patients, trainee’s reported less affiliation and more controlling behavior in comparison to their social behavior with significant others.

Lastly, we explored if psychotherapy trainees’ interpersonal behavior differs between school orientation with regard to professional and private relationships. Here, no significant differences were found in the trainees’ level of affiliation and therapeutic orientation. However, results revealed a significant interactional effect when comparing the level of interdependence within the two factors, relational setting and theoretical orientation. Individuals who attended training in CBT showed significantly more controlling behavior in professional settings, while they granted significantly more autonomy in private relationships than PD and PA trainees. For both PD groups, lower behavioral differences between private and professional relationship qualities were found.

In summary, we found that interpersonal behavior in professional rather than in private relationships could be identified to be a relevant factor to predict trainees’ WI. As presumed, high levels of affiliative interpersonal behavior in the professional relationship were associated with higher levels of HI and lower levels of SI. This result was in line with former studies, which demonstrated a positive association between introject affiliation and HI (Taubner et al., 2013). In the current study, proactive affiliation (transitive level) was defined according to Benjamin’s SASB model as affirmative, loving and protecting behavior. Affiliative reactions include disclosure behavior, love and trust toward the other person. Further it is essential to distinguish affiliation in private and professional relationships. For example, it is presumable that love toward significant others is associated with romantic emotions, whereas love in professional relationships solely describes warm and fostering behavioral attitudes of a non-romantic nature.

The present study contributed further to this result by broadening the relation to an interpersonal level. Concerning interpersonal behavior, previous studies confirmed the relevance of warmth and caring professional relationships for a successful therapy outcome (Strupp et al., 1969; Holm-Hadulla, 2000; Lambert and Barley, 2001), whereas therapists’ hostile behavior reduced the likelihood for a successful therapy outcome (Henry et al., 1986). Our present results showed that already at the beginning of psychotherapy training, trainees’ interpersonal affiliation in professional relationships seemed to be an important factor contributing to their professional development as mirrored by higher levels of HI.

No association was found between the positive developmental cycle, measured by HI, and trainees’ level of control (interdependence) in none of the relational settings. But the level of controlling interpersonal behavior in both relationships had a predictive value for SI. As expected, both variables showed negative correlations with SI. Hence, for the present data it can be summarized that SI increased when psychotherapy trainees showed more control and less affiliation in professional relationships. This result suggested that individuals who showed more controlling behavior within their patients were less engaged and more stressed with the therapeutic work they were doing (higher level of SI). This result was in line with Orlinsky and Rønnestad’s (2005) findings of therapists’ inflexibility, if pessimistic and stressed. Results could also be interpreted that trainees with higher levels of SI tended to react with controlling behavior during therapeutic sessions. For example, stress and anxieties could have evoked a trainee’s desire for a safe and controllable environment with their patients. According to Rønnestad and Skovholt’s (2003) model, trainees in the beginning of their training were especially at risk of these non-productive dynamics. However, we have to be careful in interpreting this data due to the fact that present findings display results solely derived from self-reports. Trainees’ genuine interaction with their patients remains unknown as this can only be assessed by observation. Furthermore, we have to bear in mind that participants had just started psychotherapeutic training, in which a highly reflected and accurate social behavior is expected. Thus, the trainees’ own perception might have been influenced by these aspirations. Therefore, we can only state, based on the present data analyses, that the correlations between WI and interpersonal social behavior seemed to be reasonable according to previous theoretical approaches and empirical findings, but have to be replicated by longitudinal assessment combining self-report with observer-rated measures.

Contrary to our hypothesis, significant differences between the relational settings and the trainees’ interpersonal behavior could be identified. The level of affiliation in private relationships showed significant higher values. Results could tentatively be interpreted to mean that trainees need to be able to distinguish clearly between private and professional relationships. Since a higher level of love toward significant others rather than patients is an important and reasonable differentiation to maintain clear boundaries between private and professional lives, which avoid problematic dependencies between therapist respectively trainee and patient.

The results concerning trainees’ interpersonal behavior differences between therapeutic school orientations confirmed our assumptions partially. In interdependence, significant differences between the theoretical orientations within professional and private relationships were observed as interactional effect on these two factors. As assumed earlier based on the theoretical orientations, CBT trainees differed significantly stronger between the relational settings than attendants of PA and PD trainings. Within these orientations, trainees’ private and interpersonal behavior were less differentiated. This could be explained by the fact that PD schools stress the importance of working closer to the therapist’s personality than CBT orientations are. Within each relational setting, no significant differentiations could be observed in affiliative interpersonal behavior. This result confirmed the previous finding that affiliation is an important factor for a successful psychotherapy, independent from the trainees’ respectively therapists’ theoretical orientation (Lambert and Barley, 2001).

### Implications

According to the results of the study, we stress the relevance of psychotherapeutic trainees’ social behavior in professional development. To differing extents, trainees of various theoretical orientations work close to their private social behavior. More importantly for psychotherapists’ development, seemed to be trainees’ professional interpersonal behavior, which provided strong connections to WI independent of theoretical orientation. Therefore, we understand it as reasonable to support trainees’ affiliation and autonomy-granting behavior from their very beginning of training, since our analyses represented hostile and controlling interpersonal behavior as risk factors for psychotherapeutic growth. Furthermore, we suggest raising awareness of candidates’ interpersonal – especially professional – social behavior within selection processes. For instance, a method could be chosen to evaluate candidates’ interpersonal skills in order to prevent distressing and unsatisfying training experiences. Summarizing the findings of the present study, we imply that reflections on trainees’ interpersonal behavior are relevant to understand their origin and consider their impact on the psychotherapeutic development to a sufficient extent.

### Limitations of the Current Approach

This study has only focused on psychotherapists to be in a cross-sectional design. Therefore, we plan to evaluate these findings in future longitudinal studies. As already mentioned, we used two distinctive samples to compare the interpersonal behavior in professional and private relationships. This fact could have influenced the results. However, using one sample for both settings may lead to difficulties in validity as well. Filling out the Intrex for two relation settings is problematic because of unknown retest effects and a danger of blending the two settings. The administration of an observer-based instrument could have helped to prevent those difficulties and would have produced a greater, more representative data outcome of the actual interactions between therapists, clients and significant others. Nevertheless, to evaluate an interaction it is relevant to involve the perspectives of two persons, in this case the trainees’ perception of relationship with patients’ respectively. significant others’ view. Whenever a self-report is used, it is unknown to which extent the participant might have rated the questionnaire toward social desirability. Further, we were not aware about the non-responder bias. Therefore, we cannot tell, if trainees who participated in this study were more satisfied about the psychotherapy training than the whole population.

## Conflict of Interest Statement

The authors declare that the research was conducted in the absence of any commercial or financial relationships that could be construed as a potential conflict of interest.
